# Ballistic bipolar junctions in chemically gated graphene ribbons

**DOI:** 10.1038/srep09955

**Published:** 2015-04-21

**Authors:** Jens Baringhaus, Alexander Stöhr, Stiven Forti, Ulrich Starke, Christoph Tegenkamp

**Affiliations:** 1 Institut für Festkörperphysik, Leibniz Universität Hannover, Germany; 2 Max-Planck-Institut für Festkörperforschung, Stuttgart, Germany

## Abstract

The realization of ballistic graphene pn-junctions is an essential task in order to study Klein tunneling phenomena. Here we show that intercalation of Ge under the buffer layer of pre-structured SiC-samples succeeds to make truly nano-scaled pn-junctions. By means of local tunneling spectroscopy the junction width is found to be as narrow as 5 nm which is a hundred times smaller compared to electrically gated structures. The ballistic transmission across the junction is directly proven by systematic transport measurements with a 4-tip STM. Various npn- and pnp-junctions are studied with respect to the barrier length. The pn-junctions are shown to act as polarizer and analyzer with the second junction becoming transparent in case of a fully ballistic barrier. This can be attributed to the almost full suppression of electron transmission through the junction away from normal incidence.

Immediately after the first pioneering experiments on graphene^1^,^2^,^3^ the idea of realizing Klein tunneling devices on the basis of this material was brought up^4^. A building block of this concept is the bipolar-junction which advances future electron optics, e.g. lenses and beam splitters^5^, Fabry-Pérot interferometers^6^ and which can furthermore functionalize ballistic ribbons^7^ for wave guiding^8^. Appropriate bipolar transistor structures (npn- and pnp-junctions), were proposed to be realized by manipulating locally the chemical potential such that the height of the potential barrier V_0_ exceeds the rest energy of the electrons obeying to a linear dispersion. In a purely one-dimensional scenario these relativistic electrons will fully transmit across the heterojunction irrespective of the barrier lengths, which is confined in between perfect pn-junctions^4^. Any anticipated experimental signatures of Klein tunneling, however, depend crucially on the underlying transport regimes and, in particular, on the barrier characteristics, i.e. the pn-junction *and* the barrier length D. For instance, ballistic electrons are expected to reveal an oscillating behavior of the transmission by rotating the angle of incidence with respect to the barriers^9^. Equivalently, the energy of the electrons can be varied, however, such transport experiments are difficult to realize with a simple bipolar geometry^4^. Usually, the elastic mean free path length (l_e_) is small compared to the system length scale such that the transport is globally diffusive. In this case, depending on the junctions and barriers, different scenarios are likely . For instance, in case that the barrier itself is ballistic (D ≪ l_e_) the transport measurement should comprise only the contact resistance of the first junction acting as a polarizer or momentum selector^10^,^11^,^12^.

Transport experiments across bipolar graphene junctions were realized so far by means of the electric field effect. By applying appropriately designed top gate structures, signatures of Klein tunneling were reported^13^,^9^,^14^. However, the extraction of these signatures is challenging. Electrostatic stray fields present at the edges of the gate contacts cause the pn-junctions to be typically of 1μm in size giving rise to so-called smooth junctions^13^,^10^. Such junctions usually exceed the elastic mean free path lengths of the charge carriers, i.e. the Klein tunneling effect is accompanied by diffusively scattered electrons within the junction area^13^,^9^. Furthermore, the spatial extension of the junctions restricts the experiments to low carrier concentrations (10^11^ cm^-2^) in the n- and p-type areas in order to meet the condition of short junctions compared to the wavelengths of the carriers.

In this paper we present an alternative approach. We realized ultra-narrow and well-defined potential barriers and investigated their transmission characteristics comprehensively. A controlled shift of the chemical potential within each of the electronic subsystems was achieved by Ge intercalation^15^ in lithographically patterned buffer-layer structures on SiC(0001) as sketched in Fig. 1a) (for details of the preparation we refer to the methods section). A big advantage of using epitaxially grown buffer layer structures on SiC(0001) templates is that the long-range ordering of the buffer layer is maintained after intercalation, thus transport across the junctions is not limited by disorder^16^,^17^. By means of scanning tunneling microscopy (STM), spectroscopy (STS) and local 4-tip transport the chemical potentials relative to the Dirac point were precisely measured along the 1μm-wide ribbons and correlated with transport properties. In particular we found that morphological changes to the graphene across the junctions are small so that the sublattice symmetry is preserved and K,K'-intervalley scattering is suppressed^18^. As we will demonstrate further the junctions are as small as 5 nm, i.e. they appear short for carrier concentrations up to 10^13^ cm^-2^ so that robust potential differences of up to 1 eV between the n- and p-type areas could in principle be used. We want to emphasize that the concept of chemical gating opens the possibility to measure directly the resistance of a single pn-junction without sophisticated gate structures, an important property in order to prove the proposed polarization effect of Klein tunneling barriers.

In a recent work we have demonstrated that our scanning electron microscope (SEM) reveals a convenient contrast which enables us to correlate the different intensity levels with p- or n-type doped graphene areas and, therefore, allows us to navigate and approach the nano-probes reliably to desired positions for surface sensitive transport measurements^19^. In the present study, we restrict ourselves to W=1μm wide ribbons so that electronic gaps due to confinement are negligible in these structures. Consequently, the STS spectra (shown as inset in Fig.1) show the double minima structure characteristic for intact graphene in which the Fermi energy (E_F_) is not coinciding with the Dirac point (E_D_)^20^. Upon intercalation of Ge atoms, exactly two levels of chemical potentials evolve which are directly correlated with mono- or bilayer thick Ge-films underneath^19^, c.f. Fig. 2. As for unstructured graphene, in the case of graphene nanostructures the two chemical potentials imposed by the Ge intercalation are almost symmetrically shifted with respect to the Dirac point. The electron concentration in the n-type area (n=8×10^12^ cm^-2^) is slightly higher than the hole concentration (n=6×10^12^ cm^-2^) in the p-type areas which is in perfect agreement with previous results obtained by angle resolved photoemission experiments^15^. Nonetheless, the concentrations are sufficiently low so that valley filtering due to trigonal warping effects of the Fermi surface is not relevant in our case^21^.

Careful analysis of our SEM images revealed that the process of intercalation takes place at certain positions along each of the ribbons which are likely triggered by residual defects of the SiC substrate. Although atomistic details of the intercalation process are unknown at the moment its directional dependence is important for our transport measurements: First, the intercalation taking place across the entire width of the ribbons demands the electrons to cross the barriers so that the transport properties can be correlated with the local (electronic) structure measured along the ribbons. Furthermore, the intercalated ribbon structures provide npn- and pnp- graphene junctions with variable barrier lengths D. Finally, fast diffusion in the direction across the ribbons results in comparably straight barrier fronts (width W) which is assumed in many calculations and important for the envisaged lensing effects in fully ballistic systems^6^. Based on this, the heterostructure can be viewed as an equivalent circuit of a serial connection of two tunneling junctions and a barrier in between. Details of our transport findings will be presented below.

Before the transport across bipolar structures is discussed we will concentrate first on the morphological and electronic properties of a chemically gated, single pn-junction. These characteristics of the pn-junction are decisive for the tunneling process across them. The two important parameters, i.e. the potential height V_0_ and the junction length t, can be precisely disentangled using a combination of STM and STS. Figure 2 shows both the morphology and the chemical potential taken across a junction. The chemical potential along the graphene nanostructures is extremely constant in each of the areas and not affected by corrugations within each of the graphene areas. This allows us to characterize the barriers with high accuracy. The inner potential V_0_ is determined solely by the intercalates and in case of Ge uniformly around 600 meV. As obvious, the full length of the junction (2t), deduced from the positions where the local Dirac-points change their sign, is below 5 nm and, hence, more than an order of magnitude smaller than the elastic mean free path lengths of the charge carriers. In total, this yields electric fields that are as high as F=1.2×10^6^V/cm giving rise to ultra-narrow potential steps. As we will show below, this leads to almost transparent (ballistic) junctions with low but well defined resistances R_pn—junc_. Consequences due to a lack of screening at the interface because of a vanishing density of the Dirac quasiparticles were not observed in STS, thus enhancement effects as reported for electrostatically gated junctions turn out to be not important^12^. The bending of the graphene sheet across the junction occurs on a much larger scale (≈ 10nm). Based on the fact that a long range ordered buffer layer film has been used, it is justified to assume that the AB-sublattice symmetry across the junction is maintained so that intervalley scattering for the propagating electrons at this part of the bipolar structure can be ruled out.

The transport in homogeneously doped graphene ribbons is (globally) diffuse irrespective of the type of doping. From temperature dependent measurements of the sheet resistance the mobilities μ for both n- and p-type areas were individually determined (see supplement material). At low temperatures (30 K) the carrier mobilities are in the order of 2000 cm^2^/Vs. Considering the carrier concentrations in each of the subsystems, this refers to an elastic mean free path length of l_e_ ≈ nm which is 20 times larger than the length of the Klein tunneling junction. This has interesting implications for electronic transport across heterojunctions as we will show below. First, the sheet resistances found in the n-type and p-type regimes on the ribbon are very similar and only slightly lower than those measured on epitaxial graphene^20^, i.e. different thicknesses due to Ge intercalation are irrelevant. Even more, from the STS-spectra shown above and in agreement with photoemission data^15^ we have no indications that any parasitic transport through the Ge layers takes place or that the intercalation process modifies the band structure of graphene. Therefore we conclude that the process of intercalation is very gentle as it does not destroy the unique graphene properties nor reduces the mobility as this has been found on the contrary for substitutionally doped graphene^22^.

The properties of our ultra-narrow junctions can be directly validated by transport. For this purpose all four tips were brought equidistantly in ohmic contact to the graphene ribbon, symmetrically arranged around the junction. As sketched in Fig. 3a the intertip distance is 1.5μm., i.e. the distance of the current source from the barrier is around 2 μm. We tested explicitly different distances (200 nm – 750 nm) of the inner voltage probes to the junction in order to exclude potential crosstalk effects with the barriers. Typically, the radii of the nano-contacts vary between 30 and 100 nm so we assume that the diffusively propagating electrons penetrate the barrier across its entire width W=1μm.

The IV-curves recorded at low temperature across the pn-junction are shown in Fig. 3b. For comparison also the IV-curves recorded on the n- and p-type areas are plotted which are virtually identical. The total resistance across a single pn-junction at 30 K deduced from the IV-curves is R_pn_=U/I=(162±5)Ω while the resistance in each of the subsystems (either n- or p-type) was measured to be R_n(p)_=(84(82)±4)Ω. As mentioned the IV-curves were taken in the same collinear fashion. Moreover, as the distance of the inner probes from the barrier is approximately 750nm i.e. large compared to the mean free path length, the resistance of the pn-junction itself can be calculated via R_pn-junc_=R_pn_-R_n(p)_=(78(80)±3)Ω. The resistance values given here are the mean values deduced from at least 10 junctions at different positions across the sample.

Compared to realizations with top gate structures, where the overall junction length is large compared to l_e_^14^, our resistance is much lower but realistic for ballistic Klein tunneling barriers. Generally, the resistance of a junction is inversely proportional to the transmission function T(k_y_) where k_y_=k_F_sin*Φ* is the transverse momentum of the incoming electrons (k_F_=0:037Å^-1^ is the (averaged) Fermi wave vector). Although absolute values of the transmission are linked to the spatial variation of the junction corresponding analytical expressions have been derived in the limit for sharp 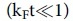 and smooth junctions 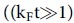 (for details see Refs.^23^,^10^). Based on our structural and electronic parameters the junction can be classified as k_F_t<1 (k_F_t≈0.7) so that the resistance is in reasonable agreement with theoretical expectations for a sharp junction 
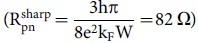
. Again, the almost perfect agreement of this estimate with our finding suggests that curvature effects of the graphene film are not significantly contributing to the resistance. The resulting transmission as a function of the incident angle *Φ* is shown in Fig. 3c) (for details see supplement). On the other hand, assuming a smooth junction the resistance would be given by 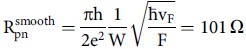 and therefore, significantly higher than the value we measured. In all these considerations the effect of defects was neglected so far. Fogler et al. developed a quantitative model to take into account also the diffusive contributions^11^. In this model, the resistance of the pn-junction is the sum of a diffusive and a ballistic contribution 
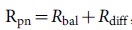
, with 
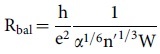
 and 
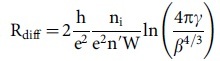
 where 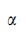 is the strength of the Coulomb interaction, 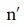 the gradient of carrier concentration, 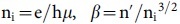 and 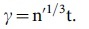 For our setup we get 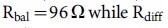 is almost zero. Despite the fact that the overall resistance we measure 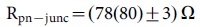 is slightly overestimated by this model, the absence of the diffusive contribution is fully in line with our interpretation of ballistic pn-junctions.

The tunneling characteristics of a *single* pn-junction can now be probed by transport across npn- and pnp-junctions with variable barrier lengths in between the two junctions. Analog to terms of optics the first pn-junction takes up the part of a polarizer while the second is the analyzer. The sample shown in Fig. 1 provides various barrier lengths (for both polarities) between 200 nm and 500 nm. The corresponding IV-curves across npn- and pnp-junctions are shown in Fig. 4. For barrier lengths around 200 nm, which is approximately 2 times the mean free path length of the carriers 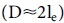, the total resistance 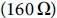 is identical to the resistance measured across a *single* pn-junction (see Fig. 4c), i.e. the resistance of the second junction is fully inapparent. Obviously, those electrons filtered by the first barrier transmit fully through the second junction. This finding can be explained only if inside the barrier scattering processes do either not take place or are limited by small-angle scattering. The barrier behaves in this length regime ballistically. The finding depends not on the polarity and is the same for npn- an pnp-junctions showing that the slight imbalance between electrons and holes in the different areas are not decisive for this effect. Even the imperfect parallelism of the two junctions (cf. SEM images in the inset of Fig. 4a) and b)) does not affect the perfect transmission through the second barrier, hence, does not lead to an increased overall resistance. For larger barriers 
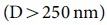
 the situation changes. As the barrier lengths increases the electrons undergo more scattering processes so that the second junction starts acting also as a polarizer whose contact resistance is measured. The barrier becomes gradually diffusive and 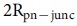 is obtained after doubling the barrier lengths.

Summarizing, we have demonstrated the formation of ballistic Klein tunneling barriers in a globally diffusive environment simply by intercalation of Ge in long-range ordered buffer layer structures grown on SiC templates. The junctions are an order of magnitude smaller than the elastic mean free path lengths and, at least, two orders of magnitude smaller than those realized via field electric effects. The insensitivity of the chemical potential regarding perturbation of the graphene film is astonishing but a key feature for further functionalization.

## Methods

We used n-doped 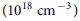 on-axis SiC(0001) Wafers from SiCrystal GmbH for sample preparation. Hydrogen etching was carried out first to remove polishing scratches and to provide a smoothly stepped surface for the subsequent preparation steps. First a buffer-layer was grown by annealing the sample at 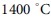 in an rf-furnace under Ar-atmosphere. The buffer layer was then patterned into 1 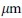 wide lines with a separation of 1 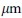 by means of reactive ion etching (RIE). The mask for the patterning process was obtained by standard UV lithography using PGMEA based photoresist and a chromium mask on a quartz glass substrate in hard-contact exposure. The etching depth was 20 nm to ensure that the buffer-layer lines are fully separated. For the reactive ion etching a mixture of 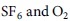 was used in a ratio of 20/7. After the etching process the intercalation of Ge was carried out in UHV by depositing five monolayers of Ge on the buffer layer using a Knudsen cell. Subsequently, the sample was annealed at a temperature of 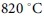 to induce the intercalation process.

All measurements were performed under ultra-high vacuum (UHV) conditions with an Omicron 4-point probe STM system combined with a SEM (type Gemini). The SEM images were taken with an accelerating voltage of 15 kV and a probe current of 1 nA. STM images were obtained with one of the STM tips with a bias of 1.2 V and a tunneling current of 500 pA. The bias and tunneling current were also used as set point for the tunneling spectroscopy. Tunneling spectra were recorded using lock-in technique with a modulation voltage of 1200 Hz and an amplitude of 19 mV.

Transport data across pn- and npn/pnp-junctions were recorded in a linear 4-point probe configuration with the outer tips as current source and the inner tips as voltage probes while the pn-junctions were always located between the inner voltage probes. The contacting of tips and sample was carried out separately for each tip. All tips were first brought into tunneling contact with the sample, then moved to the desired position. Thereafter, the feedback loop was turned off and the tips were approached to the sample surface in nm-steps while checking the contact resistances. Once a stable configuration was reached the IV-measurements were performed by ramping the current from -10 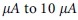 and measuring the corresponding voltages.

## Author Contributions

C.T. and U.S. conceived and designed the experiments, J.B. performed the transport and STM experiments, A.S. and S.F. produced and characterized the samples, C.T. wrote the paper. All authors reviewed the manuscript.

## Supplementary Material

Supplementary Information Supplementary figures

## Figures and Tables

**Figure 1:**
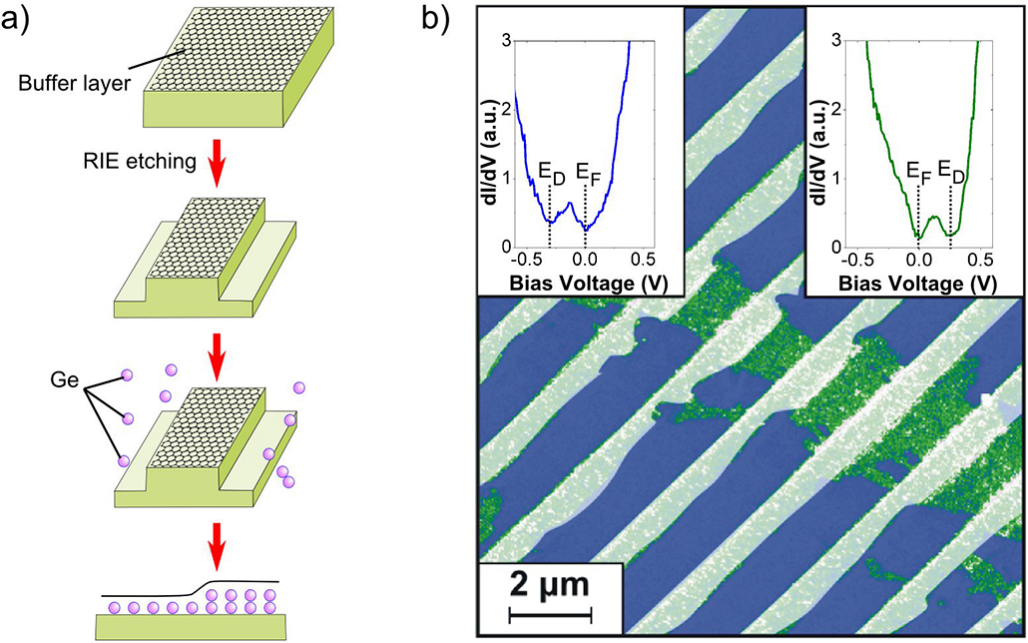
Functionalization of the buffer layer to receive bipolar graphene ribbons. a) Schematic of the different processes (formation of the buffer layer, etching, and intercalation) in order to reveal graphene nanostructures of defined width W with spatially different chemical potentials. b) Color-coded SEM image. The green (blue) colors denote p-type (n-type) graphene areas. The local chemical potentials are deduced from local spectroscopy (STS) curves shown as insets.

**Figure 2:**
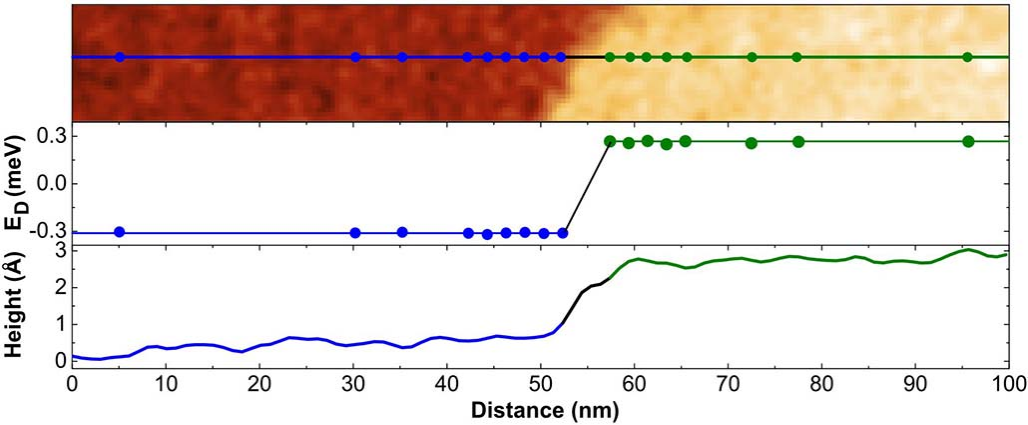
Structure and chemical potential across a pn-junction. From top to down: STM image (+1.2 V, 0.5 nA, L=1.5μm) of a pn-junction. Local morphology and chemical potential across a single pn-junction. The height profile across the junction clearly reveals that the p-type graphene (green) is higher by around 0.24 nm. The dots are deduced from spatially resolved STS measurements taken in the center of the ribbon similar to those shown in Fig. 1. The built-in potential is around 600 meV and the pn-junction length 2t < 5nm.

**Figure 3:**
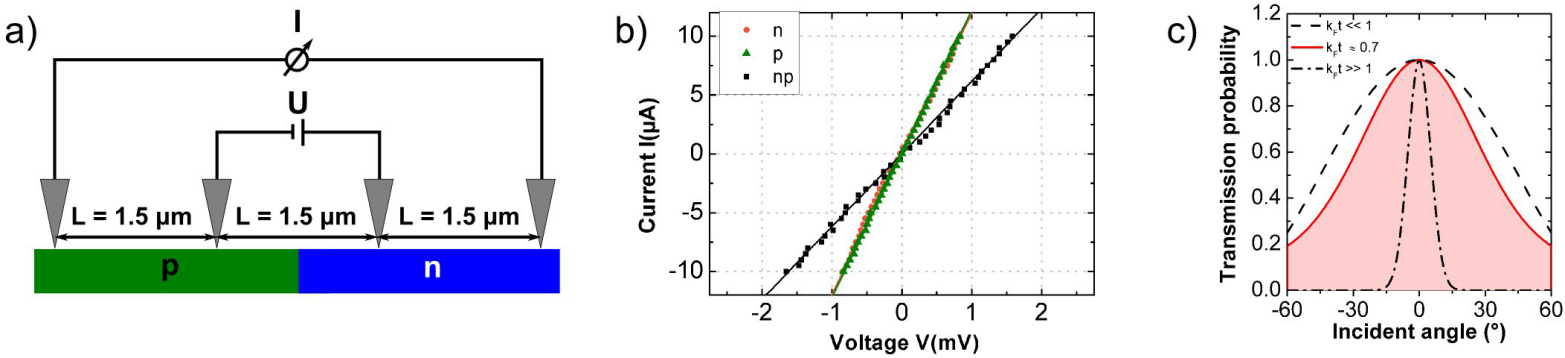
Local transport across a single pn-junction. a) Schematic collinear tip assembly used for the transport measurements on the graphene ribbons. The equidistant spacing between the tips was L= 1μm. b) IV-measurement across a pn-junction at T = 30 K. As reference, also the IV-curves measured on n- and p-type samples are shown. c) Plot of the transmission probability for propagating electrons as a function of the angle of incidence (0° means perpendicular).

**Figure 4:**
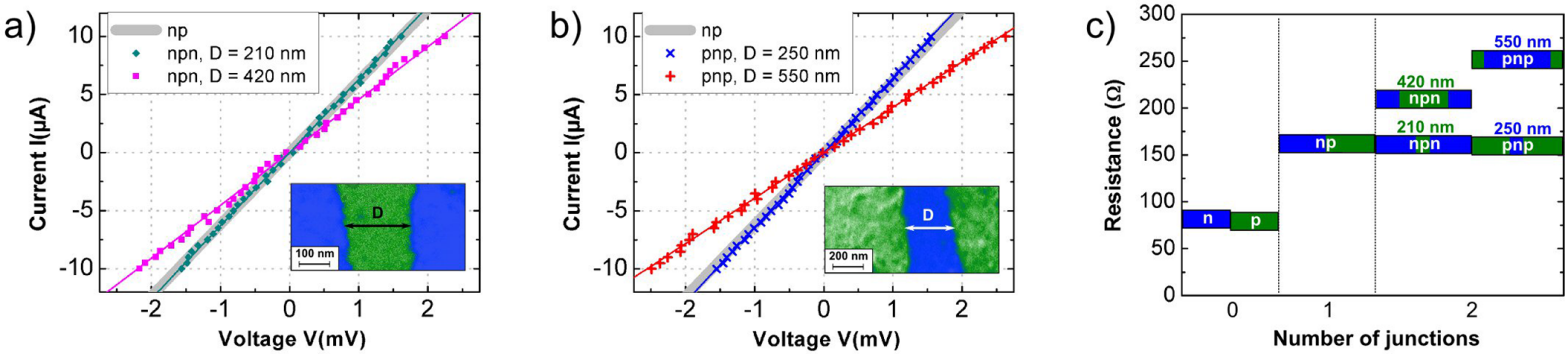
Transport across npn- and pnp- junctions. IV-curves across a) npn- and b) pnp-junctions for different barrier lengths D. The insets show corresponding SEM images of the junctions. For the short barrier lengths the IV-curves fit perfectly into the gray-shaded areas, which represent the values measured across a *s*ingle pn-junction (T = 30 K). c) Summary of the resistances measured for 0, 1, and 2 pn-junctions in 1μm.-wide graphene nanoribbons. For the latter two different barrier regimes are shown. For details see text.
