# Supplementary material for: Ballistic bipolar junctions in chemically gated graphene ribbons
Source: Sci Rep. 2015 Apr 21;5:9955. doi: 10.1038/srep09955 (PMC4404713; doi:10.1038/srep09955)
Supplement: Supplementary Information — Supplementary figures [file srep09955-s1.pdf]

Ballistic bipolar junctions in chemically gated graphene ribbons

Jens Baringhaus[1], Alexander Stöhr[2], Stiven Forti[2],

Ulrich Starke[2], and Christoph Tegenkamp[1*]

[1] *Institut für Festkörperphysik, Leibniz Universität Hannover, Germany and*

[2] *Max-Planck-Institut für Festkörperforschung, Stuttgart, Germany*

(Dated: February 3, 2015)

PACS numbers:

*Electronic address: `tegenkamp@fkp.uni-hannover.de`

I. CALCULATION OF THE TRANSMISSION PROBABILITY

The calculation of the transmission probability is carried out for electrons with an energy E which is half of the potential step ($E = \frac{V_0}{2} = 300\ meV$). In our case this assumption is sufficiently close to our experimental conditions since the difference in the chemical potential of the n- and p-type doped areas is comparably small (about 50 meV). In figure S1 the transmission probabilities for smooth and sharp potential steps are plotted against the incident angle. In the case of a sharp potential step ($k_F t << 1$) the transmission probability is given by $T = \cos^2(\phi)$ [1, 2]. As obvious, a preference for normal incidence is given by this transmission function. However, the transmission probability is relatively high for all angles except those close to 90°. In the case of a smooth potential step ($k_F t >> 1$) the transmission probability is $T = \exp(-\pi k_F t \sin^2(\phi))$ [1, 2]. As for the sharp potential step $T(0) = 1$, but decreases rapidly to zero for larger angles in contrast to the sharp potential step. This is the reason for the strong collimation effect of a smooth potential step, basically no other particles than those with an incidence angle close to 0 are transmitted.

In our experimental setup, the width of the potential step is in the transition region between a sharp and a smooth step since $k_F t \approx 0.7$. The corresponding transmission probability is calculated under the assumption that the formula given above for the smooth potential step is still valid. The result is shown in figure S1 and reflects the transitional character between a smooth and a sharp potential step.

II. SHEET RESISTANCE AND CARRIER MOBILITIES

In order to discriminate the contribution of the pn junctions from the background, the resistances of entirely p- and n-doped graphene areas have been measured. For this purpose all four tips are placed on a p-doped or n-doped area in a linear arrangement with equal probe spacings. The sheet resistances are deduced from measurements in dual configuration, i.e two resistances are measured with different voltage/current probe geometries. The two configurations used here are schematically shown in fig. S2a). Following the van der Pauw equations the sheet resistance R_S, and the resistances in the two configurations shown in fig. S2a) are related by the expression

$$\exp(\frac{2\pi R_A}{R_S}) - \exp(\frac{2\pi R_B}{R_S}) = 1\ .$$

Hence, from the two resistances R_A and R_B, the sheet resistance can be directly calculated without the need for any geometrical correction factors [3].

The IV-curves recorded exhibit linear (ohmic) behavior and the corresponding resistance is deduced by a linear fit. In fig. S2b) the sheet resistances of a p-doped and an n-doped area with respect to the probe spacing are shown exemplarily for a measurement taken at room temperature. No variation of the sheet resistance with the probe spacing is observed for neither of the two doping levels, a typical signature for two dimensional transport behavior. The mean sheet resistance of the p-doped area (782 Ω) is slightly higher than on the n-doped area (729 Ω). The temperature dependence of the sheet resistance is rather weak as can be seen in fig. S2c). Both areas show an almost identical small decrease of the sheet resistance with decreasing temperature. The sheet resistance of the p-doped area is decreasing to a minimum value of 502 Ω at 32 K while the minimum value for the n-doped area is slightly higher (520 Ω at 32 K). The temperature dependence can be described by a simple model taking into account scattering processes with surface phonons [4] as obvious from the fit in fig. S2c). The corresponding mobilities are extracted using the Drude model where

$$\mu = \frac{1}{enR_\mathrm{S}}.$$

The carrier concentration n is deduced from

$$n = \int_0^\infty dE \frac{4}{2\pi(\hbar v_F)^2} |E| f(E)$$

where v_F is the Fermi-velocity and f(E) the Fermi-Dirac distribution. The mobilities obtained by this model are also shown in fig. S2c). Overall, the p-doped area exhibits higher mobilities than the n-doped area with a maximum value of 2762 cm^2/Vs at 32 K (1714 cm^2/Vs for the n-doped area).

---

[1] Cheianov, V. V. & Falko, V. Selective transmission of Dirac electrons and ballistic magnetoresistance of n-p junctions in graphene. *Phys. Rev. B* **74**, 041403 (2006).

[2] Allain P. & Fuchs, J. Klein tunneling in graphene: optics with massless electrons. *Eur. Phys. J. B.* **83**, 301-317 (2011).

[3] Wang, F. et al. Sensitivity study of micro four-point probe measurements on small samples *J. Vac. Technol. B* **28**, 34-40 (2010)

[4] Chen, J.-H., Jang, C., Xiao, S., Ishigami, M. & Fuhrer, M. S. Intrinsic and extrinsic performance

limits of graphene devices on SiO2. *Nature Nanotechnology* **3**, 206-209 (2008)

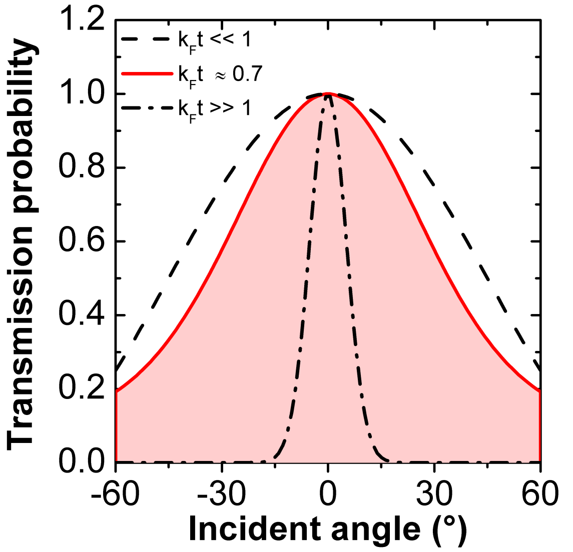

FIG. S1: Transmission probability T as a function of the incidence angle across a potential step for an electron energy of half the potential step. Three cases are shown: a sharp potential step ($k_F t << 1$), a smooth step ($k_F t >> 1$) and an intermediate case ($k_F t \approx 0.7$). The formulas for each step type are given in the main text.

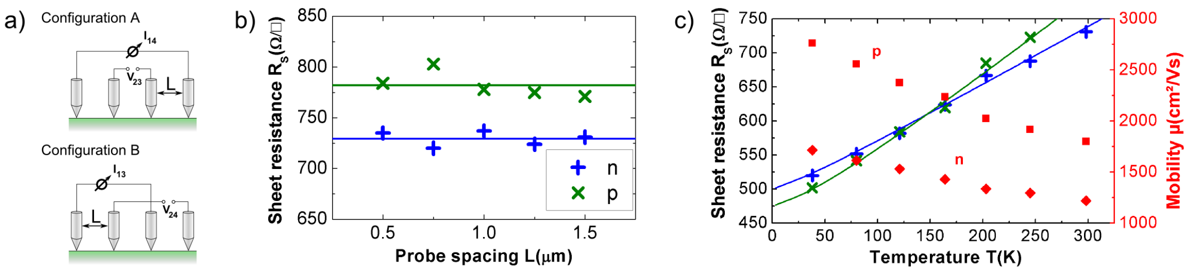

FIG. S2: a) The two setups used for the dual configuration measurements to deduce R_A and R_B. b) Sheet resistance of n and p doped areas as a function of probe spacing at room temperature. c) Sheet resistances and mobilities of n and p doped graphene areas as a function of temperature.
